# Outcomes of Low Vision Rehabilitation Programs for Children: A Prospective Observational Cohort Study

**DOI:** 10.3390/children13081030

**Published:** 2026-08-02

**Authors:** Areej Okasheh-Otoom

**Affiliations:** Faculty of Applied Medical Sciences, Jordan University of Science and Technology, P.O. Box 3030, Irbid 22110, Jordan; aaokashah@just.edu.jo

**Keywords:** low vision rehabilitation, pediatric visual impairment, electronic low vision aids, functional vision, visual rehabilitation

## Abstract

**Highlights:**

**What are the main findings?**

- Multidisciplinary low vision rehabilitation significantly improved visual, functional, educational, and mobility outcomes in children.
- Electronic low vision aids provided greater improvements in near visual acuity and reading performance than non-electronic low vision aids.

**What is the implication of the main finding?**

- Comprehensive paediatric low vision rehabilitation supports improved functional independence and educational participation in children with visual impairment.
- Electronic low vision aids may provide additional benefits for near visual function and reading performance when incorporated into multidisciplinary rehabilitation.

**Abstract:**

Purpose: To evaluate the visual, functional, educational, and mobility outcomes of a comprehensive pediatric low vision rehabilitation (LVR) program and determine whether electronic low vision aids (LVAs) provide additional benefit. Methods: A multicenter prospective observational cohort study was conducted across three tertiary low vision rehabilitation centers in Jordan. Two hundred children aged 6–16 years with moderate-to-severe visual impairment were enrolled. The intervention group (*n* = 150) received comprehensive multidisciplinary rehabilitation, including optical LVAs, rehabilitation training, educational support, and electronic LVAs when indicated, while the comparison group (*n* = 50) received routine low vision care without participation in the structured multidisciplinary rehabilitation program. Primary outcomes were LogMAR distance visual acuity (VA) and LV Prasad Functional Vision Questionnaire (FVQ) scores. Secondary outcomes included near VA, reading speed, mathematics performance, and mobility independence. Results: At 12 months, the intervention group demonstrated significant improvement in distance VA from 0.87 ± 0.13 to 0.65 ± 0.11 LogMAR (mean change −0.22; 95% confidence interval [CI], −0.25 to −0.19; *p* < 0.001), whereas the comparison group showed only minimal improvement. FVQ scores increased significantly from 42.3 ± 8.7 to 76.5 ± 9.1 (+34.2 points; *p* < 0.001). Within the intervention group, reading speed improved by 25.3 words/minute, mathematics scores by 15.7%, and mobility independence by 32% (all *p* < 0.001), whereas only minimal changes were observed in the comparison group. Multivariate regression analysis identified electronic LVA use as the strongest independent predictor of functional improvement (β = 0.41, *p* < 0.001). Conclusions: Comprehensive pediatric low vision rehabilitation improved visual, educational, and mobility outcomes. In an exploratory subgroup analysis, children using electronic low vision aids demonstrated greater functional improvement than those using non-electronic low vision aids.

## 1. Introduction

Childhood visual impairment remains a major global public health challenge, disproportionately affecting children in low- and middle-income countries (LMICs), where access to specialized ophthalmic and rehabilitation services is often limited [1,2]. Although many causes of childhood blindness are preventable or treatable, a considerable proportion of children experience irreversible visual impairment requiring long-term rehabilitative intervention rather than curative treatment [2,3]. In these children, reduced visual function may substantially impair educational attainment, psychosocial development, mobility independence, and quality of life [4].

In the Eastern Mediterranean Region, uncorrected refractive error, amblyopia, retinal disorders, corneal opacity, and cataract remain major causes of childhood visual impairment [4,5]. Retinopathy of prematurity remains an important cause of childhood visual impairment worldwide, particularly in middle-income countries where neonatal survival has improved substantially [5]. Jordan has witnessed progressive development in pediatric ophthalmic care over the past decade; however, structured low vision rehabilitation services remain inconsistently distributed across the region and are frequently concentrated within tertiary referral centers. Access to assistive technologies and specialized rehabilitation programs remains limited, particularly for school-aged children requiring long-term educational support [1,2,4,6].

Visual impairment during childhood affects multiple developmental domains extending beyond visual acuity alone [7]. Difficulties in reading fluency, visuomotor coordination, environmental navigation, classroom participation, and social interaction may lead to reduced academic achievement and long-term social dependency [8,9]. Consequently, modern pediatric low vision rehabilitation aims not only to improve visual task performance but also to optimize functional independence and participation in daily life. Delayed access to rehabilitation services may adversely affect developmental and psychosocial outcomes in children with vision loss [8]. Cerebral visual impairment has emerged as one of the leading causes of childhood visual impairment in many developed countries owing to improved survival of premature infants and children with neurological disorders [9].

Comprehensive LVR typically combines optical devices, environmental modifications, orientation and mobility training, educational adaptations, and assistive technologies to maximize functional vision and participation in daily activities [10,11,12]. Previous studies conducted predominantly in high-income countries have demonstrated that structured rehabilitation programs improve reading efficiency, near-task performance, and vision-related quality of life [12,13,14]. Nevertheless, evidence from LMIC settings remains comparatively limited, particularly regarding long-term pediatric outcomes and educational performance [15].

Electronic LVAs, including portable video magnifiers, closed-circuit television systems, digital magnification software, and optical character recognition devices, have emerged as important adjuncts to conventional optical rehabilitation [14,16]. Unlike traditional magnifiers, electronic LVAs provide adjustable magnification, contrast enhancement, brightness control, and text-to-speech functionality, potentially enabling more sustained visual task performance in educational environments. Several studies have suggested that assistive technologies and structured rehabilitation interventions may improve reading performance, functional vision, and participation among individuals with low vision [13,14,15,16]. However, access to these technologies in developing countries remains restricted because of financial and infrastructural barriers.

Despite increasing recognition of the importance of pediatric rehabilitation, there remains a lack of large-scale multicenter studies evaluating low vision rehabilitation outcomes in Jordan and across the Eastern Mediterranean Region. Furthermore, limited data are available regarding the specific contribution of electronic LVAs to functional and educational outcomes among visually impaired children in LMIC contexts.

Therefore, the present study aimed to evaluate the visual, functional, academic, and mobility outcomes of a comprehensive pediatric low vision rehabilitation program conducted across multiple centers in Jordan. In addition, the study sought to determine whether the incorporation of electronic low vision aids provided additional benefit beyond conventional rehabilitation alone.

## 2. Methods

### 2.1. Study Design and Setting

This prospective observational cohort study was conducted across three tertiary low vision rehabilitation centers in Jordan. Participants were enrolled between January and December 2024 and were followed for 12 months after enrolment. The study followed the Strengthening the Reporting of Observational Studies in Epidemiology (STROBE) guidelines and was designed to evaluate the effectiveness of a comprehensive pediatric low vision rehabilitation program in children with moderate-to-severe visual impairment.

The participating centers represented major referral facilities providing specialized pediatric low vision services in Jordan and served children from both urban and semi-urban regions. Standardized rehabilitation protocols and assessment procedures were implemented across all participating centers to ensure consistency of intervention delivery and outcome evaluation.

The study adhered to the principles of the Declaration of Helsinki and received approval from the Institutional Review Board of Jordan University of Science and Technology (IRB No. 680). Written informed consent was obtained from parents or legal guardians prior to enrolment. Assent was additionally obtained from participating children whenever appropriate according to age and cognitive ability.

### 2.2. Sample Size Calculation

Sample size estimation was performed using G*Power version 3.1 (Heinrich Heine University, Düsseldorf, Germany). Based on a moderate expected effect size (Cohen’s d = 0.5), an alpha level of 0.05, and statistical power of 90% for within-subject comparisons between baseline and follow-up assessments, the minimum required sample size was calculated as 172 participants. To compensate for an anticipated attrition rate of approximately 15%, a total sample of 200 children was recruited.

### 2.3. Participants

Children aged 6–16 years diagnosed with moderate-to-severe visual impairment according to World Health Organization criteria were eligible for inclusion. Participants were recruited consecutively from the participating rehabilitation centers during routine clinical referral.

### 2.4. Inclusion Criteria

Children were included if they:

▪ Had stable visual impairment not amenable to further medical or surgical treatment;
▪ Demonstrated sufficient cognitive ability to complete functional and educational assessments;
▪ Were enrolled in formal educational programs;
▪ Were willing to participate in scheduled rehabilitation sessions and follow-up assessments.

### 2.5. Exclusion Criteria

Children were excluded if they:

▪ Had acute ophthalmic disease requiring ongoing medical intervention;
▪ Had severe neurological or developmental disorders significantly limiting participation in rehabilitation activities;
▪ Had previous structured low vision rehabilitation within the preceding 12 months;
▪ Failed to complete baseline assessments.

A total of 217 children were screened for eligibility, of whom 200 met the inclusion criteria and were enrolled. Participants were categorized into either a comprehensive rehabilitation group (*n* = 150) or a comparison group (*n* = 50) according to routine clinical care pathways rather than random assignment.

This study was designed as a prospective observational cohort study rather than a randomized controlled trial. Assignment reflected routine clinical care pathways within participating rehabilitation centers. Children who received the structured multidisciplinary rehabilitation program constituted the intervention cohort, whereas children receiving routine low vision care, including refractive correction and conventional low vision management, without participation in the structured multidisciplinary rehabilitation program, served as the comparison group. The study aimed to evaluate real-world rehabilitation outcomes under routine clinical practice conditions.

Allocation to the intervention or comparison group was determined according to routine clinical care pathways at the participating rehabilitation centers rather than random assignment. Children referred to and enrolled in the structured multidisciplinary rehabilitation program were included in the intervention group, whereas those receiving routine low vision care without participation in the structured rehabilitation program constituted the comparison group. Within the intervention group, the prescription of electronic or non-electronic low vision aids was individualized based on clinical assessment, taking into account factors such as baseline visual function, visual demands, age, educational requirements, manual dexterity, cognitive ability, anticipated compliance, device availability, affordability, and clinician judgment. No single clinical characteristic alone determined device allocation.

Children in the intervention group received a structured multidisciplinary rehabilitation program consisting of functional assessment, low vision aid prescription, orientation and mobility training, educational adaptation, and parental counselling. Children in the comparison group received routine low vision care, including refractive correction and conventional low vision management, without participation in the structured multidisciplinary rehabilitation program. The study did not withhold treatment; rather, it compared outcomes between children receiving routine care and those enrolled in the comprehensive rehabilitation program.

Within the intervention group, the selection of electronic or non-electronic low vision aids was individualized according to routine clinical practice and based on a comprehensive clinical assessment. Factors considered included the severity of visual impairment, near visual demands, educational requirements, age, cognitive ability, manual dexterity, anticipated compliance, device availability, affordability, and clinician judgment. The primary analysis compared the intervention group with the comparison group. In addition, a prespecified secondary subgroup analysis was conducted within the intervention cohort to compare outcomes between children prescribed electronic LVAs (*n* = 94) and those prescribed non-electronic LVAs (*n* = 56).

During the 12-month follow-up period, nine participants (4.5%) were lost to follow-up, including six from the intervention group and three from the comparison group. Sensitivity analyses were performed using last observation carried forward for missing data.

### 2.6. Baseline Clinical Characteristics

Baseline demographic and clinical characteristics were recorded for all participants before initiation of the rehabilitation program.

### 2.7. Rehabilitation Intervention

The comprehensive rehabilitation program consisted of three sequential phases: detailed functional assessment, individualized device prescription, and structured rehabilitation training.

### 2.8. Comprehensive Functional Assessment

All participants underwent standardised ophthalmic and functional evaluation at baseline and follow-up visits. Assessments included:

▪ Distance VA using LogMAR charts;
▪ Near VA using M notation;
▪ Contrast sensitivity assessment using the Pelli–Robson chart;
▪ Functional vision assessment using the LV Prasad FVQ;
▪ Educational performance testing;
▪ Mobility evaluation.

### 2.9. Optical and Electronic LVAs Prescription

Optical rehabilitation devices included handheld magnifiers (3×–7×), dome magnifiers, stand magnifiers, and spectacle-mounted magnification systems. Device selection was individualized according to visual demands, age, and educational requirements.

Electronic LVAs included portable electronic magnifiers, closed-circuit television (CCTV) systems, tablet-based digital magnification software, and optical character recognition (OCR) reading devices. Electronic LVA usage was defined as utilization during at least four school-related sessions per week throughout the rehabilitation period.

### 2.10. Rehabilitation Training

Structured rehabilitation training included:

▪ Eight orientation and mobility sessions;
▪ Six low vision device training sessions;
▪ Teacher-oriented educational adaptation sessions;
▪ Parental counselling and home-environment guidance.

Training sessions were delivered by multidisciplinary rehabilitation teams consisting of optometrists, low vision specialists, mobility trainers, and educational support personnel.

### 2.11. Functional Vision Assessment

Functional vision was evaluated using a modified version of the LV Prasad Functional Vision Questionnaire (FVQ) adapted for pediatric low vision assessment [17]. The questionnaire consisted of 16 items evaluating four domains:

▪ Near-vision activities;
▪ Distance-vision activities;
▪ Activities of daily living;
▪ Mobility-related tasks.

Responses were graded using a three-point scale:

▪ 0 = unable to perform;
▪ 1 = able to perform with difficulty;
▪ 2 = able to perform independently.

Total scores were converted to a percentage scale ranging from 0 to 100, with higher scores indicating better functional visual performance. Internal consistency of the questionnaire in the present study was high (Cronbach’s α = 0.91).

### 2.12. Academic Assessments

Academic performance was evaluated using standardized educational assessments appropriate for participant age and school grade. Outcome measures included:

▪ Reading comprehension accuracy (% correct responses);
▪ Reading speed (words per minute wpm);
▪ Mathematics performance (% standardised score).

All educational assessments were conducted under standardised lighting and viewing conditions.

Reading performance was assessed using age-appropriate standardized reading passages. Reading speed was recorded as the number of correctly read words per minute (wpm). Mathematics performance was assessed using curriculum-based standardized mathematics tests appropriate for the participant’s educational level and reported as percentage scores.

### 2.13. Mobility Assessment

Mobility independence was assessed using a structured five-item mobility rating scale evaluating:

▪ Indoor navigation;
▪ Outdoor mobility;
▪ Obstacle avoidance;
▪ Stair negotiation; and;
▪ Independent classroom mobility.

Each item was scored on a 3-point scale ranging from 0 to 2, with higher scores indicating greater independence.

### 2.14. Statistical Analysis

Statistical analyses were performed using IBM SPSS Statistics version 29 (IBM Corp., Armonk, NY, USA). Continuous variables were presented as mean ± standard deviation (SD), while categorical variables were presented as frequencies and percentages.

Within-group comparisons were analyzed using paired *t*-tests, whereas between-group comparisons were assessed using independent *t*-tests. Repeated-measures analysis of variance (ANOVA) was used to evaluate changes over time. Multiple linear regression analysis was performed to identify predictors of functional improvement following rehabilitation.

Effect sizes were calculated using Cohen’s d, and 95% confidence intervals (CIs) were reported where appropriate. Multicollinearity within regression models was assessed using variance inflation factor (VIF) analysis. Statistical significance was defined as *p* < 0.05.

## 3. Results

### 3.1. Participant Flow and Follow-Up

A total of 217 children were screened for eligibility during the study period. Of these, 200 participants met the inclusion criteria and were enrolled in the study, including 150 children in the intervention group and 50 in the comparison group. During the 12-month follow-up period, nine participants (4.5%) were lost to follow-up, including six from the intervention group and three from the comparison group. The overall study completion rate was 95.5%. A post hoc power analysis based on the final analyzed sample sizes demonstrated adequate statistical power for the primary between-group comparison between the intervention and comparison groups (achieved power = 94.6%).

**Figure 1:**
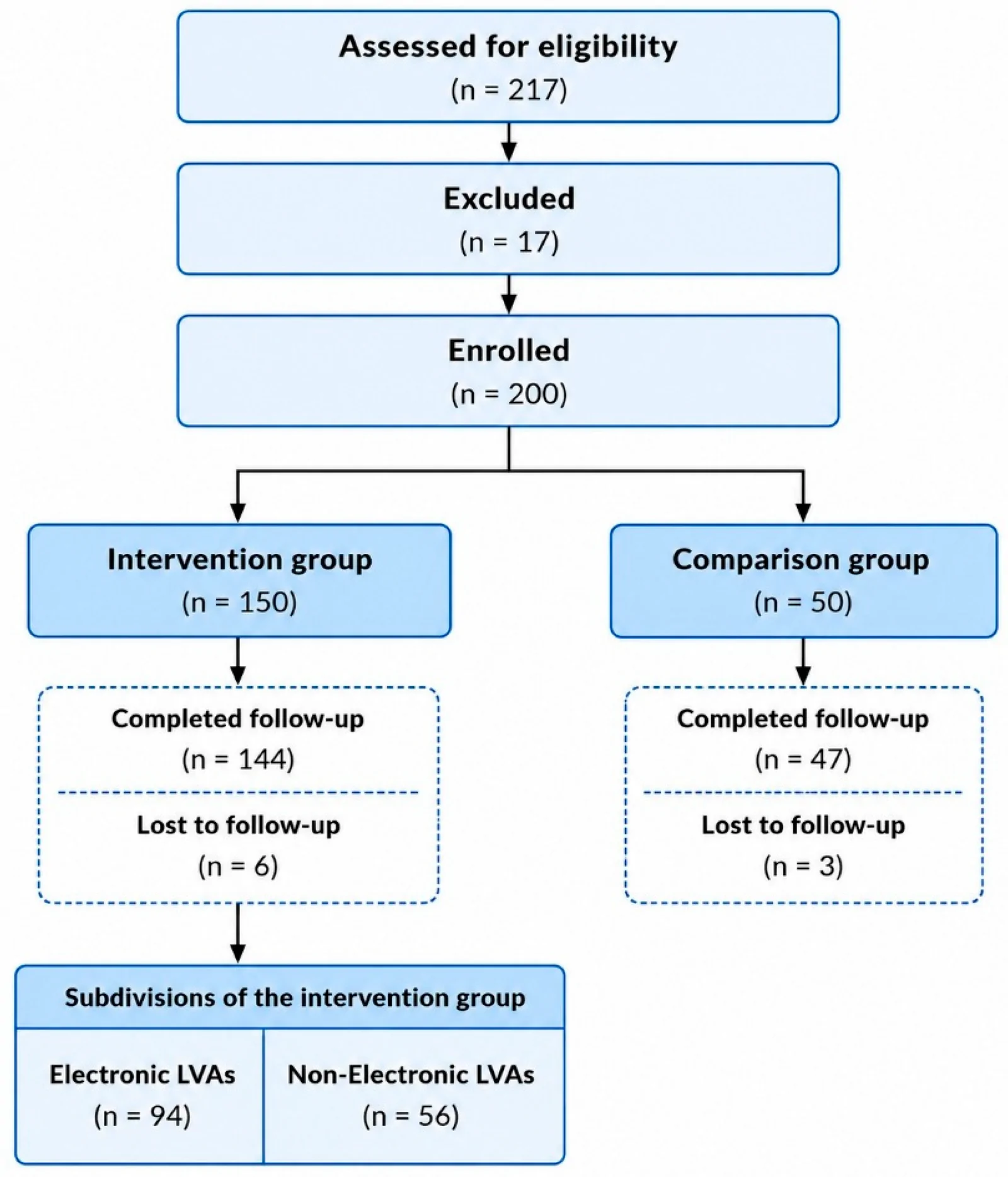
Flow diagram illustrating participant screening, enrollment, allocation to the intervention and comparison groups, follow-up, and subdivision of the intervention cohort into the Electronic LVA and Non-Electronic LVA subgroups.

Missing follow-up data were handled using last observation carried forward. Sensitivity analyses demonstrated no material change in the direction or statistical significance of the study findings. Attendance records were maintained for all rehabilitation sessions. Rehabilitation adherence was calculated as the percentage of scheduled sessions attended during the study period. Participants attending at least 80% of scheduled sessions were considered adherent to the rehabilitation program. A participant flow diagram is presented in Figure 1.

### 3.2. Baseline Demographic and Clinical Characteristics

Baseline demographic and clinical characteristics are summarized in Table 1. No statistically significant differences were identified between the intervention and comparison groups at baseline (*p* > 0.05 for all comparisons), indicating adequate comparability between groups prior to rehabilitation.

The mean participant age was 10.4 ± 2.9 years, and 54% of participants were male. Albinism represented the most common underlying diagnosis (28%), followed by retinopathy of prematurity (22%), congenital cataract (18%), inherited retinal disorders (15%), optic nerve disorders (10%), and miscellaneous causes (7%).

Mean baseline distance visual acuity was 0.87 ± 0.13 LogMAR in the intervention group and 0.89 ± 0.14 LogMAR in comparison group. Baseline FVQ scores were similarly comparable between groups.

### 3.3. Visual Acuity Outcomes

#### 3.3.1. Distance Visual Acuity

Significant improvement in distance visual acuity was observed in the intervention group following rehabilitation. Mean distance visual acuity improved from 0.87 ± 0.13 LogMAR at baseline to 0.65 ± 0.11 LogMAR at 12 months, corresponding to a mean improvement of −0.22 LogMAR (95% confidence interval [CI], −0.25 to −0.19; *p* < 0.001) and a large effect size (Cohen’s d = 0.84).

In contrast, the comparison group demonstrated only minimal improvement during follow-up, with mean visual acuity improving from 0.89 ± 0.14 to 0.83 ± 0.13 LogMAR. Between-group comparison at 12 months demonstrated a statistically significant difference favoring the intervention group (*p* < 0.001).

**Figure 2:**
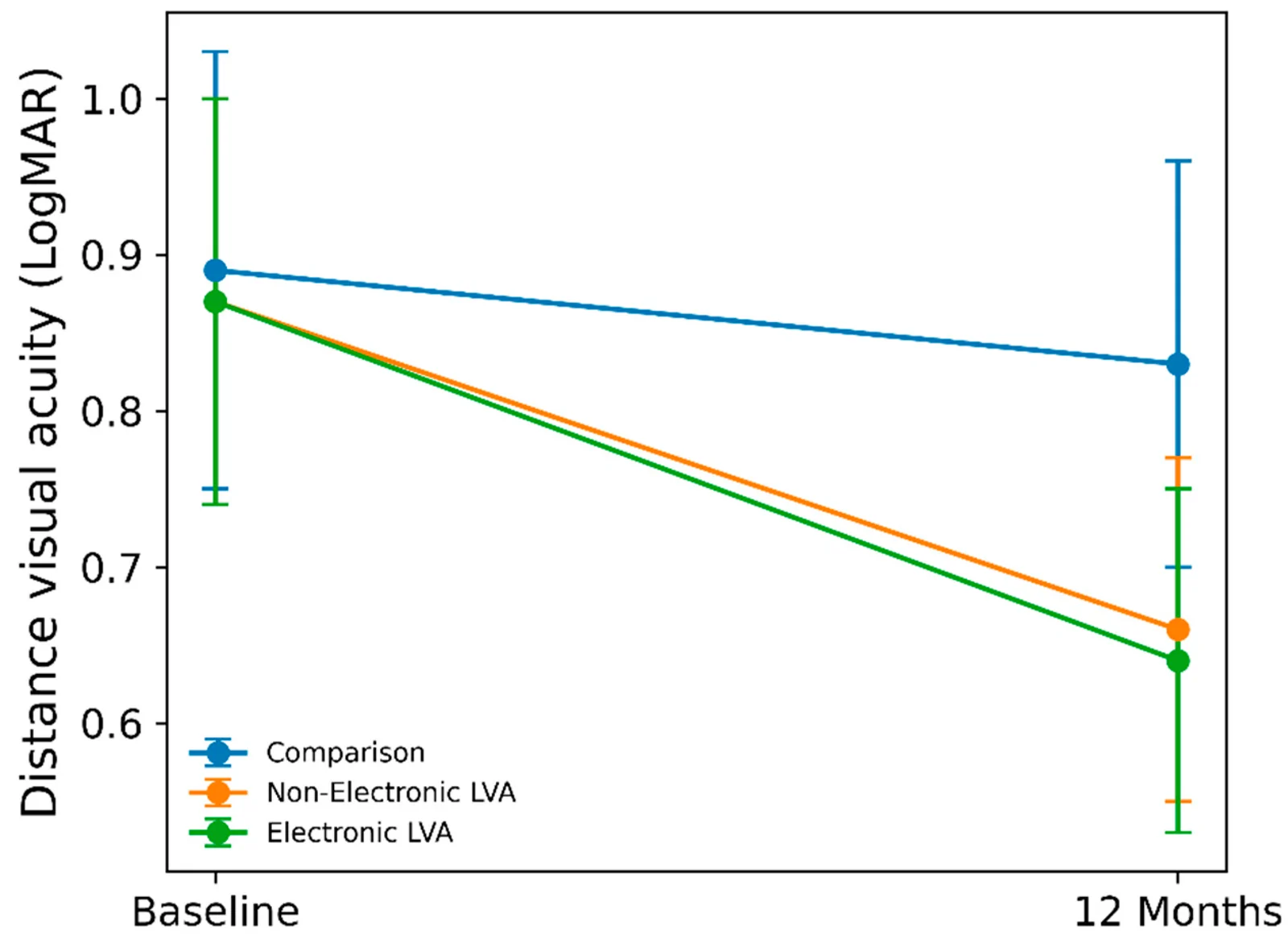
Changes in distance visual acuity (LogMAR) from baseline to the 12-month follow-up in the comparison group and the intervention subgroups (non-electronic and electronic low vision aids). Lower LogMAR values indicate better visual acuity. Error bars represent standard deviations (SDs).

Repeated-measures ANOVA demonstrated a significant interaction between treatment group and time (F = 41.2, *p* < 0.001), indicating significantly greater improvement in the intervention group over the follow-up period. Changes in distance visual acuity are illustrated in Figure 2.

#### 3.3.2. Near Visual Acuity

**Figure 3:**
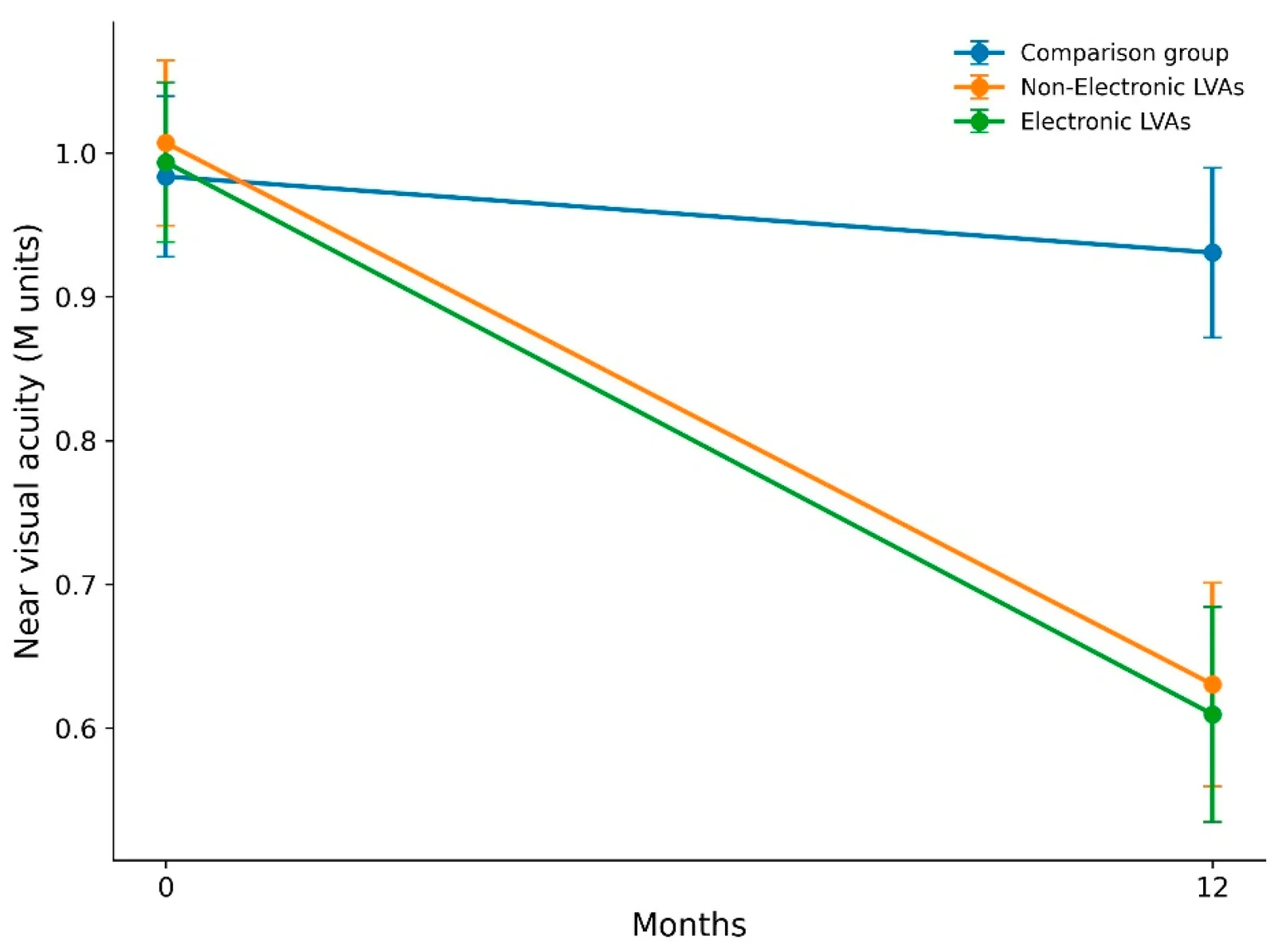
Changes in near visual acuity from baseline to the 12-month follow-up in the comparison group and the intervention subgroups (non-electronic and electronic low vision aids). Lower values indicate better near visual acuity. Error bars represent standard deviations (SDs).

Near visual acuity improved significantly in the intervention group, whereas only minimal change was observed in the comparison group. Mean near visual acuity improved from 1.00 ± 0.06 M at baseline to 0.62 ± 0.07 M at 12 months in the intervention group, whereas only minimal change was observed in the comparison group (0.98 ± 0.06 M to 0.93 ± 0.06 M). Children using Electronic LVAs demonstrated greater improvement in near visual acuity than participants using Non-Electronic LVAs (*p* < 0.001). Figure 3 presents changes in near visual acuity.

#### 3.3.3. Functional Vision Outcomes

Substantial improvement in functional vision was observed in the intervention group, whereas only minimal improvement was observed in the comparison group. Mean FVQ scores increased from 42.3 ± 8.7 at baseline to 76.5 ± 9.1 at 12 months in the intervention group, representing a mean increase of 34.2 points (*p* < 0.001).

Improvement was observed across all FVQ domains, including near activities, distance activities, daily living tasks, and mobility-related activities. The greatest gains were observed in educational near tasks and independent classroom functioning.

**Figure 4:**
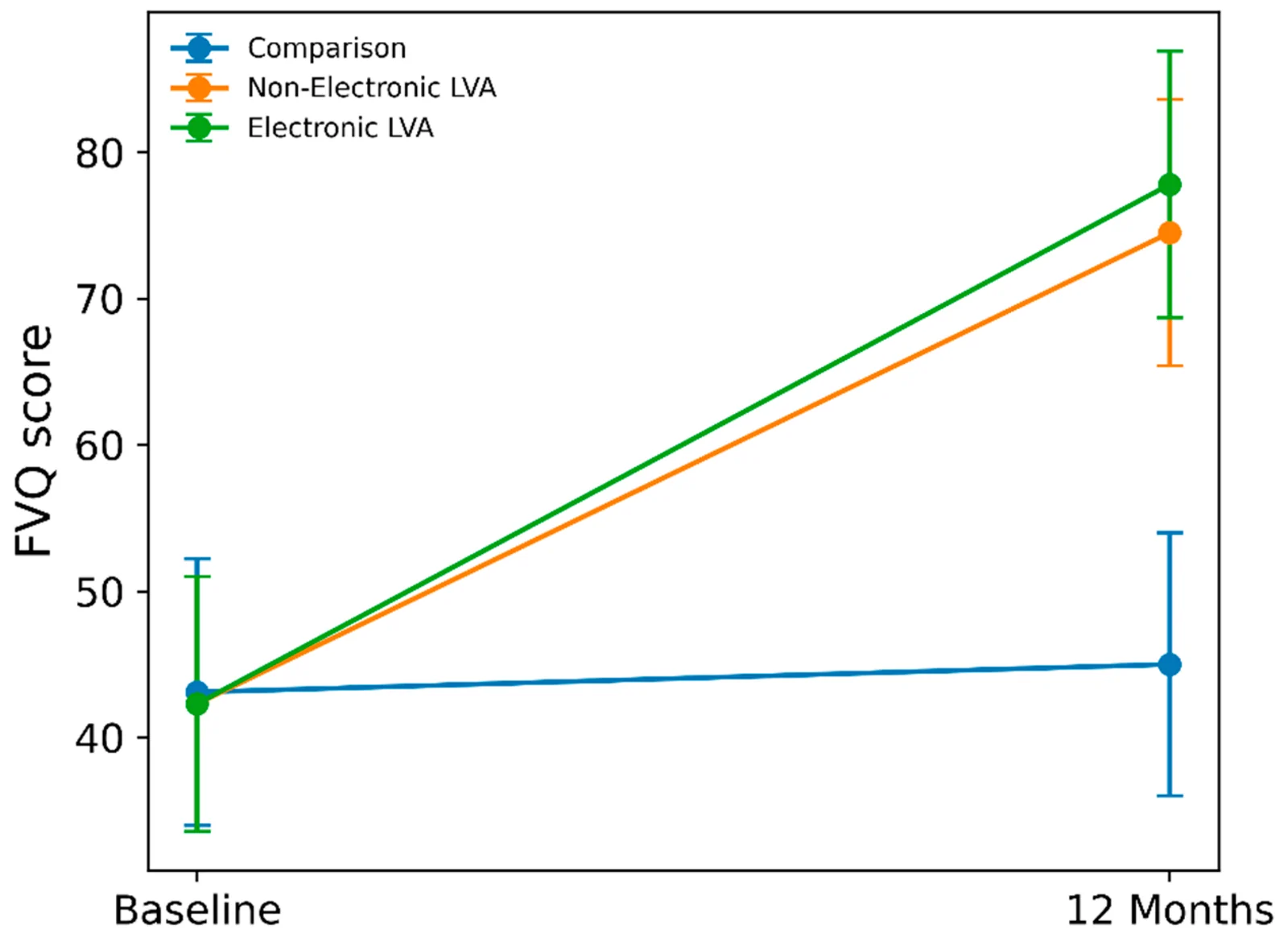
Changes in Functional Vision Questionnaire (FVQ) scores from baseline to the 12-month follow-up in the comparison group and the intervention subgroups (non-electronic and electronic low vision aids). Higher FVQ scores indicate better functional vision. Error bars represent standard deviations (SDs).

In contrast, the comparison group demonstrated only minimal improvement in FVQ scores during follow-up. Changes in functional vision are illustrated in Figure 4.

### 3.4. Academic Performance Outcomes

#### 3.4.1. Reading Speed

Reading speed improved significantly in the intervention group compared with the comparison group Within the intervention cohort, children using electronic low vision aids demonstrated significantly greater improvement in reading speed than those using non-electronic low vision aids. Mean reading speed increased from 38.1 ± 9.4 words/minute at baseline to 63.4 ± 10.2 words/minute at 12 months in the intervention group, corresponding to a mean increase of 25.3 words/minute (*p* < 0.001).

**Figure 5:**
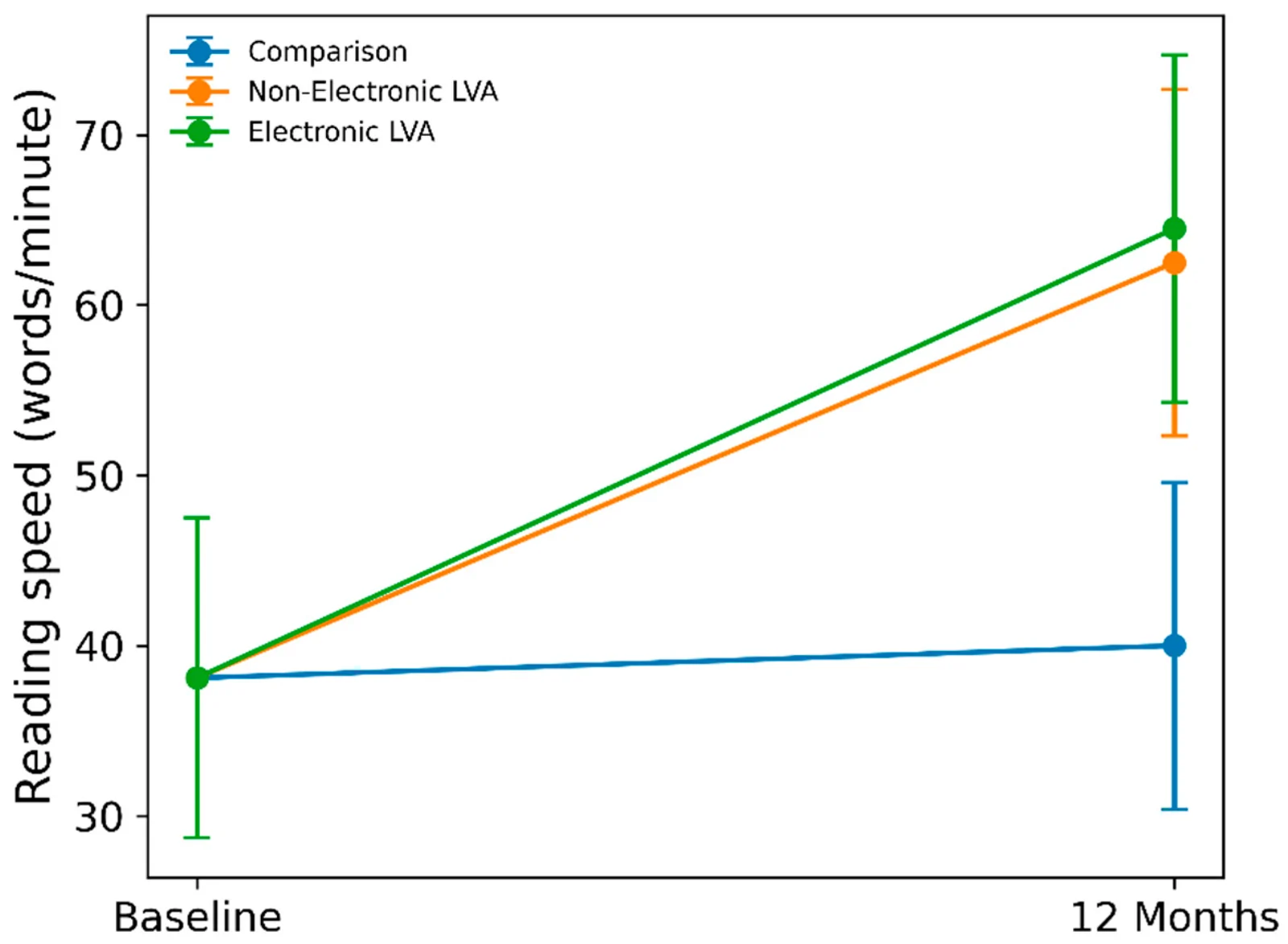
Changes in reading speed from baseline to the 12-month follow-up in the comparison group and the intervention subgroups (non-electronic and electronic low vision aids). Error bars represent standard deviations (SDs).

Among children receiving rehabilitation, those using electronic low vision aids demonstrated significantly greater improvement in reading speed than those receiving conventional low vision aids without electronic devices. Figure 5 illustrates baseline and 12-month reading speed for the comparison group and for the Electronic LVA and Non-Electronic LVA subgroups within the intervention group.

#### 3.4.2. Reading Comprehension and Mathematics Performance

Reading comprehension accuracy improved significantly in the intervention group from 58.7 ± 10.1% at baseline to 74.5 ± 9.6% at follow-up, corresponding to a mean increase of 15.8 percentage points (*p* < 0.001). Participants demonstrated enhanced sustained reading ability and reduced visual fatigue during educational tasks.

Mathematics performance improved from 52.4 ± 11.3% at baseline to 68.1 ± 10.4% at follow-up, corresponding to a mean increase of 15.7 percentage points (*p* < 0.001). Improvements were significantly greater than those observed in the comparison group.

### 3.5. Mobility Outcomes

Mobility independence improved significantly in the intervention group compared with the comparison group. Mean mobility independence scores increased from 41.2 ± 8.5 at baseline to 73.2 ± 9.0 at 12 months, corresponding to a mean improvement of 32.0 points (*p* < 0.001). The greatest improvements were observed in obstacle avoidance, stair negotiation, and independent classroom mobility.

### 3.6. Secondary Subgroup Analysis Within the Intervention Cohort

A subgroup analysis was performed among participants receiving comprehensive rehabilitation to compare outcomes between children using electronic low vision aids and those not using electronic low vision aids. Children using electronic low vision aids achieved significantly greater improvements in several outcome domains, including near visual acuity, functional vision, and reading performance.

### 3.7. Electronic LVA Users Demonstrated:

▪ Greater improvement in near visual acuity;
▪ Higher FVQ gains;
▪ Faster reading speeds;
▪ Greater task endurance during educational activities.

The mean FVQ improvement among electronic LVA users exceeded that observed among non-electronic LVA users by 12.7 percentage points (*p* < 0.001). The overall changes in visual, functional, educational, and mobility outcomes are summarized in Table 2.

### 3.8. Multivariate Regression Analysis

Multiple linear regression analysis was performed to identify independent predictors of functional improvement following rehabilitation. Variables entered into the model included age, baseline visual acuity, diagnosis category, socioeconomic status, rehabilitation adherence, and electronic LVA usage.

Electronic LVA usage emerged as the strongest independent predictor of FVQ improvement (β = 0.41, *p* < 0.001). Better baseline visual acuity (β = 0.19, *p* = 0.008) and higher socioeconomic status (β = 0.12, *p* = 0.03) were also independently associated with greater functional improvement.

The final regression model demonstrated good explanatory performance with acceptable multicollinearity indices (all variance inflation factor values < 2.0). The results of the multivariable linear regression analysis are summarized in Table 3.

## 4. Discussion

The present multicenter study demonstrated that comprehensive pediatric LVR was associated with substantial improvements in visual function, educational performance, and mobility independence among children with moderate-to-severe visual impairment in Jordan. Significant gains were observed across multiple outcome domains, including distance visual acuity, functional vision, reading performance, mathematics achievement, and independent mobility. Importantly, in the exploratory subgroup analysis, children using electronic LVAs achieved greater functional improvement than participants using non-electronic LVAs.

The observed improvement in distance visual acuity following rehabilitation is consistent with previous evidence demonstrating the effectiveness of structured low vision interventions in pediatric populations [12,13,18,19]. Although rehabilitation does not reverse the underlying ocular pathology, optimization of residual visual function through appropriate optical correction and assistive technology may substantially improve task performance and visual efficiency. The magnitude of improvement observed in the present study was clinically meaningful and was accompanied by significant gains in functional visual performance.

One of the most important findings of this study was the marked improvement in FVQ scores following rehabilitation. Functional vision increased by more than 34 percentage points over the 12-month follow-up period, indicating substantial enhancement in the ability to perform educational, mobility-related, and daily living activities independently. These findings support previous reports demonstrating that functional rehabilitation may have greater real-world relevance than isolated visual acuity measures alone [10,11,17]. Improvements in classroom participation, sustained reading, and independent navigation suggest that rehabilitation contributed not only to visual task performance but also to broader participation in educational and social environments. Similar observations have been reported in studies examining functional and motor rehabilitation outcomes among children with low vision [20].

The educational improvements observed in the present study are particularly noteworthy. Reading speed improved significantly following rehabilitation, especially among children using electronic LVAs. Sustained reading performance represents a major challenge for children with visual impairment because conventional optical magnifiers frequently require reduced working distances and prolonged visual effort. Electronic magnification systems provide adjustable magnification, contrast enhancement, and improved ergonomics, potentially reducing visual fatigue during prolonged educational activities [14,16]. The significant increase in mathematics performance further suggests that rehabilitation may positively influence broader academic engagement beyond reading-related tasks alone.

Participants who used electronic low vision aids achieved greater functional and educational outcomes compared with participants who did not use electronic low vision aids, supporting previous evidence regarding the benefits of electronic assistive technologies for visually impaired children [14,16]. In the present study, electronic LVA usage independently predicted functional improvement even after adjustment for baseline visual acuity and socioeconomic variables. This finding suggests that electronic assistive technologies may provide additional benefit. Portable electronic magnifiers and digital accessibility tools may be particularly valuable in educational settings where visual demands are dynamic and prolonged. Because this comparison was performed within the intervention cohort and treatment allocation was not randomized, these subgroup findings should be interpreted as exploratory rather than confirmatory and should not be considered evidence of a causal relationship. In addition, selection bias and residual confounding cannot be excluded because device allocation was based on clinical indication.

The present findings are especially relevant within resource-constrained developing-country settings, where access to pediatric rehabilitation services and assistive technologies remains limited. Previous studies from resource-limited settings have demonstrated poorer educational and functional outcomes among children with untreated low vision [15]. In Jordan and across the Eastern Mediterranean Region, low vision rehabilitation remains inconsistently integrated into routine pediatric ophthalmic care [6,15,21]. Financial barriers, limited specialist availability, insufficient teacher training, and restricted access to assistive technologies continue to impede rehabilitation delivery.

The integration of multidisciplinary rehabilitation approaches may have contributed substantially to the favorable outcomes observed in this study. In addition to device prescription, participants received orientation and mobility training, teacher-directed educational adaptation, and parental counselling. This holistic rehabilitation model likely enhanced adherence and facilitated the transfer of visual skills into daily academic and mobility activities. Previous evidence suggests that combined educational and mobility interventions improve long-term participation outcomes among visually impaired children [18,19,21,22,23,24].

The study also demonstrated a high retention rate, with more than 95% of enrolled participants completing follow-up assessments. This may reflect strong family engagement and the perceived benefit of rehabilitation services. Furthermore, the multicenter design enhances the generalizability of findings within Jordanian tertiary rehabilitation settings.

A notable strength of the present study was the 12-month follow-up period, which enabled evaluation of medium-term rehabilitation outcomes beyond the immediate effects of device prescription.

Several limitations should be acknowledged. First, the study was non-randomized, which may introduce selection bias despite comparable baseline characteristics between groups. In addition, the sample size calculation was based on the primary within-group longitudinal analysis rather than between-group comparisons. Therefore, comparisons involving the electronic and non-electronic LVA intervention subgroups should be interpreted as exploratory because subgroup allocation was based on clinical indication rather than randomization. Consequently, unequal subgroup sizes, potential selection bias, and residual confounding may have influenced the observed differences. Second, the majority of participants were recruited from urban tertiary centers, potentially limiting generalizability to rural populations with reduced access to specialized rehabilitation services. Third, although the follow-up period was sufficient to evaluate medium-term rehabilitation outcomes, longer follow-up may be necessary to assess sustained educational achievement and long-term independence. Finally, socioeconomic and educational environmental factors may have influenced rehabilitation outcomes despite adjustment within multivariate analyses. Although baseline characteristics were comparable and multivariable analyses were used to adjust for measured confounders, the possibility of residual confounding due to unmeasured variables cannot be entirely excluded.

Despite these limitations, the study provides important evidence supporting the effectiveness of comprehensive pediatric low vision rehabilitation in a developing-country context. This study represents a large multicenter evaluation of pediatric low vision rehabilitation outcomes in the Eastern Mediterranean Region and among the few studies examining the additional contribution of electronic LVAs within resource-constrained settings. Future studies should evaluate long-term educational attainment, psychosocial outcomes, cost-effectiveness, and accessibility of rehabilitation services in broader regional populations. Further investigation into school-based rehabilitation integration and tele-rehabilitation approaches may also be valuable, particularly in underserved areas.

Overall, the findings of the present study support the incorporation of structured low vision rehabilitation and electronic assistive technologies into national pediatric ophthalmic and educational support programs. Early rehabilitation intervention may substantially improve functional independence, educational participation, and quality of life among visually impaired children [18,19,24].

## 5. Conclusions

Comprehensive pediatric low vision rehabilitation was associated with significant improvements in visual function, educational performance, and mobility independence among children with moderate-to-severe visual impairment in Jordan. Rehabilitation resulted in clinically meaningful gains in distance visual acuity, functional vision, reading performance, and academic achievement over the 12-month follow-up period.

Exploratory subgroup analyses suggested that children using electronic low vision aids demonstrated greater improvements in several functional and educational outcomes than those using conventional low vision aids alone.

The findings of this multicenter study support the integration of structured low vision rehabilitation services into routine pediatric ophthalmic care in resource-constrained healthcare settings. In addition, wider accessibility to electronic assistive devices, multidisciplinary rehabilitation services, and school-based educational support may substantially improve participation, independence, and quality of life among visually impaired children.

Further multicenter studies with longer follow-up periods are warranted to evaluate long-term educational, psychosocial, and economic outcomes associated with pediatric low vision rehabilitation and assistive technology implementation.

## Figures and Tables

**Table 1:** Baseline demographic and clinical characteristics of study participants.

Characteristic	Intervention Group (*n* = 150)	Comparison Group (*n* = 50)	*p* Value
Age (years), mean ± SD	10.5 ± 2.8	10.2 ± 3.0	0.58
Male sex, *n* (%)	81 (54.0)	27 (54.0)	0.99
Urban residence, *n* (%)	104 (69.3)	32 (64.0)	0.47
Baseline distance VA (LogMAR), mean ± SD	0.87 ± 0.13	0.89 ± 0.14	0.41
Baseline FVQ score, mean ± SD	42.3 ± 8.7	43.1 ± 9.1	0.55
Albinism, *n* (%)	42 (28.0)	14 (28.0)	0.99
Retinopathy of prematurity, *n* (%)	33 (22.0)	11 (22.0)	0.99
Congenital cataract, *n* (%)	27 (18.0)	9 (18.0)	0.99
Inherited retinal disorders, *n* (%)	23 (15.3)	7 (14.0)	0.82
Optic nerve disorders, *n* (%)	15 (10.0)	5 (10.0)	0.99
Other diagnoses, *n* (%)	10 (6.7)	4 (8.0)	0.74

Functional Vision Questionnaire (FVQ); standard deviation (SD); visual acuity (VA).

**Table 2:** Changes in visual, functional, educational, and mobility outcomes following rehabilitation.

Outcome Measure	Baseline	12 Months	Mean Change	*p* Value
Distance VA (LogMAR)	0.87 ± 0.13	0.65 ± 0.11	−0.22	<0.001
Near visual acuity (M)	1.00 ± 0.06	0.62 ± 0.07	−0.38	<0.001
FVQ score (%)	42.3 ± 8.7	76.5 ± 9.1	+34.2	<0.001
Reading speed (words/minute)	38.1 ± 9.4	63.4 ± 10.2	+25.3	<0.001
Reading comprehension (%)	58.7 ± 10.1	74.5 ± 9.6	+15.8	<0.001
Mathematics performance (%)	52.4 ± 11.3	68.1 ± 10.4	+15.7	<0.001
Mobility independence score	41.2 ± 8.5	73.2 ± 9.0	+32.0	<0.001

Functional Vision Questionnaire (FVQ); standard deviation (SD); visual acuity (VA). Data are presented as mean ± SD unless otherwise indicated.

**Table 3:** Multivariable linear regression analysis identifying predictors of functional vision improvement following rehabilitation.

Variable	β Coefficient	95% CI	*p* Value
Electronic low vision aid usage	0.41	0.28 to 0.54	<0.001
Baseline visual acuity	0.19	0.05 to 0.33	0.008
Socioeconomic status	0.12	0.02 to 0.22	0.030
Age	−0.07	−0.15 to 0.01	0.090
Rehabilitation adherence	0.18	0.06 to 0.30	0.004

Dependent variable: Change in Functional Vision Questionnaire score. Confidence interval (CI).

## Data Availability

The data presented in this study are available from the corresponding author upon reasonable request. The data are not publicly available because they contain information that could compromise participant privacy.
